# Madness of the crowd: Understanding mass behaviors through a multidisciplinary lens

**DOI:** 10.3389/fpsyg.2022.924511

**Published:** 2022-08-19

**Authors:** Emily Brindal, Naomi Kakoschke, Andrew Reeson, David Evans

**Affiliations:** ^1^Health & Biosecurity, Commonwealth Scientific and Industrial Research Organization, Adelaide, SA, Australia; ^2^Data61, Commonwealth Scientific and Industrial Research Organization, Canberra, ACT, Australia

**Keywords:** crowd behavior, social norms, panic buying, conspiracy theories, health protective behaviors, modeling, social networks, conformity

## Abstract

Mass or crowd behaviors refer to those that occur at a group level and suggest that crowds behave differently to individuals. Mass behaviors are typically triggered by a significant societal event. The ongoing COVID-19 pandemic has provided many tangible examples of crowd behaviors that have been observed globally, suggesting possible common underlying drivers. It is important to provide a deeper understanding of such behaviors to develop mitigation strategies for future population-level challenges. To gain deeper insight into a variety of crowd behaviors, we perform a conceptual analysis of crowd behaviors using three detailed case studies covering observable behavior (panic buying and health protective actions) and mass beliefs (conspiracy theories) that have resulted or shifted throughout the pandemic. The aim of this review was to explored key triggers, psychological drivers, and possible mitigation strategies through a mixture of theory and published literature. Finally, we create experimental mathematical models to support each case study and to illustrate the effects of manipulating key behavioral factors. Overall, our analyses identified several commonalties across the case studies and revealed the importance of Social Identity Theory and concepts of trust, social connection, and stress.

## Introduction

### What are mass behaviors?

Mass or crowd behaviors are referred to in many ways including mob psychology, swarm behavior, collective behavior, and herding. All capture the idea of looking at groups of people *en masse*, and how crowds behave differently from individuals. The ongoing COVID-19 pandemic provides many tangible examples of crowd behaviors such as panic buying, non-compliance with mask wearing, and growing levels of conspiracy theorizing. These behavioral examples have been witnessed globally suggesting an underlying human element or driver. Having a deeper understanding of these mass actions and what prompted them can assist in mitigating and harnessing them for future population challenges including new health pandemics, natural disasters, and ongoing crises such as climate change.

There are multiple theoretical explanations for mass behaviors which originate from different fields of research. It appears that most are triggered by large, significant societal events both immediate and extended. Underlying individual and psychological as well as social and cultural factors are also likely to be important. Psychological triggers such as conformity or following others could have counterintuitive impacts for one behavior compared to another (for example, conformity could conceivably promote charitable donations as well as panic buying). It is unclear what explanations are likely to be the most parsimonious.

### The current study

In attempt to gain insight into a variety of crowd behaviors, this paper explores triggers, underlying drivers and possible mitigation strategies using detailed case studies of observable behaviors (i.e., panic buying, health protective behaviors) and attitudes (i.e., conspiracy theories) that occur at a mass level. These conceptual reviews will be based on psychological and economics theory and published research, and evaluated through conceptual experimental mathematical models that aim to illustrate effects of manipulating key behavioral factors identified.

## Conceptual review: Case studies

### Case study 1: Panic buying - wiping out the competition

We define panic buying as an outbreak of individuals rushing to purchase more than necessary for their immediate needs. It is rushed rather than planned, driven in part by fear of impending shortages. Panic buying was observed widely in Australia during its first national lockdown. According to Australian Bureau of Statistics data, at the announcement of lockdown monthly spending doubled on toilet paper, rice, pasta and flour (News, 2020). A recent systematic review suggested that panic buying behavior will change over the course of the pandemic (Billore and Anisimova, 2021).

#### Theoretical explanations for panic buying

**Figure 1:**
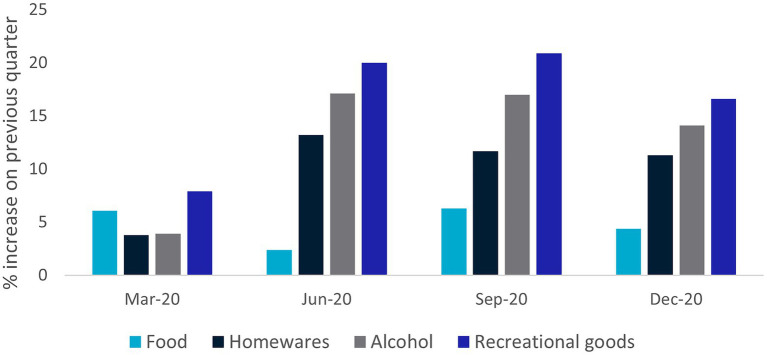
Growth in household spending on goods, by category, Australia 2020. Source: Australian Bureau of Statistics, Insights into household consumption, December quarter 2020 3/03/2021.

Proposed lockdowns and restrictions to movement are recognized as significant triggers for panic buying (Kim and Tandoc, 2021). Panic buying has also been associated with government announcements pre-COVID (Prentice, 2017). Thus, panic buying appears to be a “normal” reaction to impending shortages of products, or restrictions on activities more generally (Yuen et al., 2020). In the absence of being able to control the pandemic, and associated restrictions, individuals may turn to things which they can control, such as shopping (Zeng et al., 2020). It is notable that spending on most categories of goods, not just “essentials,” grew very strongly in 2020 (Figure 1). Indeed, research showed that individuals’ self-protection efficacy predicted panic-buying and product consumption during the COVID-19 pandemic (Tabernero et al., 2020). Spending on ‘services’ such as travel and eating out also fell substantially, leaving many households with more to spend on other things and able to express their control through shopping.

Social media also provided an unprecedented, real-time glimpse into people’s behaviors around COVID-19 announcements increasing visibility and social information about other people’s behavior. One qualitative study suggested that social media increased awareness of stock unavailability in supermarkets which lead people to act proactively by stockpiling or panic buying (Naeem and Ozuem, 2021). Similarly, another study used Twitter data, namely, tweets related to panic buying of toilet paper and found that negative content was the most influential and widespread (Leung et al., 2021). Thus, social media platforms may facilitate the spreading of content *en masse* which leads to fear and in turn, panic buying.

Panic buying, particularly of toilet paper, is highly visible and likely to also trigger behaviors. Conversely not panic buying is not visible to others, so it is likely that its prevalence is exaggerated. Studies have shown that scarcity predicted increased panic buying during the COVID-19 pandemic, and the previous SARS pandemic (Chan and Koh, 2006; Rayburn et al., 2021). Regret is a more powerful motivator than positive emotions (Kahneman and Tversky, 1982), so purchases may be made to avoid the prospect of future regret of missing out. Shelf scarcity alone can also alter consumer behavior (Robinson et al., 2016). One study was able to increase the purchase of high-priced items (compared to low and middle) from 5 to 40% of purchases by manipulating shelf scarcity. Toilet paper takes up a lot of shelf space and is clearly vacant when stocks are depleted. This suggests that simple visual clues, even in times with no panic, are enough to alter behavior.

At the collective level panic buying is irrational because it means that resources are being stockpiled by individuals rather than shared. This is not necessarily true at the individual level. If there is an expectation that others will panic buy it becomes a self-fulfilling prophecy as individuals strive to beat the crowd. Game Theory suggests it is hard to stop such behavior. Even if one person considers it irrational, they may participate if they believe (correctly or incorrectly) others are sufficiently irrational to participate; even the belief (correct or incorrect) that others may believe (correctly or incorrectly), that others are irrational may be sufficient to trigger the behavior. Dynamic models of crowd behavior show that collective behavior can rapidly flip to irrational (Granovetter, 1978). Rational individuals could also be concerned about information asymmetries; even if you think it is irrational (i.e., there should be plenty of toilet paper), if others are doing it then they may know something you do not (Banerjee, 1992).

In one sense panic buying can be construed as selfish and motivated by self-interest. Interestingly, happiness is more likely to make people act in selfish ways than sadness (Tan and Forgas, 2010), a feeling in short supply during the spread of the pandemic. Yet, a theorized outcome of negative emotions is an attentional bias toward external rather than internal processing which ultimately makes people more sensitive to social and visible cues (Fiedler, 2001). Taken together, it is possible that panic buying is not driven by purely selfish motives, but by over-sensitivity to social cues thus highlighting the potential importance of social psychological theory.

Social Identity Theory (SIT; Tajfel, 1978) describes that as a society we operate in varying social groups. Some groups form our ingroup, or the one we belong to, and others become outgroups. Perceptions of outgroups are usually less favorable and individual motivations will focus on protecting and strengthening the ingroup. At face value SIT suggests, a global, outside threat should trigger ingroup protection at a national level. Yet, panic buying is not in the best interest of the wider community. Lockdown may change the immediate perception of the ingroup from the broader community to those in a person’s immediate living circumstance. Actions such as stockpiling are of benefit to a person’s new, micro ingroup.

Social norms (Sherif, 1936) are likely to be a significant contributor to panic buying. These are communicated through the actions we see others taking and visibility cues such as shelf scarcity. Feldman (1984) suggested that in addition to assisting with biological survival, social norms allow expression of shared value and the avoidance of embarrassing behaviors and improve predictability of our social interactions. Norms are most salient in interactions high in ambiguity (Hetherington et al., 2006). In the instance of a global pandemic and lockdown – a situation high in ambiguity about the “right” thing to do, people are likely to become reliant on social cues. This may also be emphasized by the experience of a negative mood state and irrationality promoted by observing other people’s behavior. Visible cues that communicate social information are likely to account for panic buying across multiple theoretical considerations. A supermarket carpark full of cars, and multiple news/social media stories about panic buying, communicate to the population that this is a ‘normal’ behavior that most people are doing which then promotes others to do this regardless of whether they view it as rational.

#### Why toilet paper? Modeling panic buying

Here, we hypothesize that heterogeneous agents following a simple social learning proces causes some products to be more susceptible to panic buying (e.g., toilet paper) than others (e.g., pharmaceuticals). To test this hypothesis, we develop a model in which each agent starts panic buying a product if they observe sufficient other agents panic buying the product. This heuristic reflects that consumers are likely to follow social norms and respond to visual cues in making decisions, as discussed in section “Theoretical explanations for panic buying”. In the model, we let the agents vary in their propensities to panic buy a given product. We also let the visibility of panic buying vary between different products, with the agents having a greater propensity to panic buy higher visibility products.

Formally, to model the process of panic buying, we first set the product’s visibility level v > 0. We then draw the consumers’ panic thresholds randomly from a normal distribution, as in Granovetter (1978), and scale each consumer’s threshold by $\frac{1}{v}$. Therefore, consumers’ panic thresholds are smaller for higher visibility products (larger v). Finally, we simulate the panic buying process. Here, we parameterize the system such that in the initial state of panic (e.g., a lockdown scare), about 10% of consumers are triggered to panic buy the product (have a threshold of zero or less). Other consumers then observe this initial panic buying and those with low thresholds start to panic buy. This process repeats and pushes the system to equilibrium.

**Figure 2:**
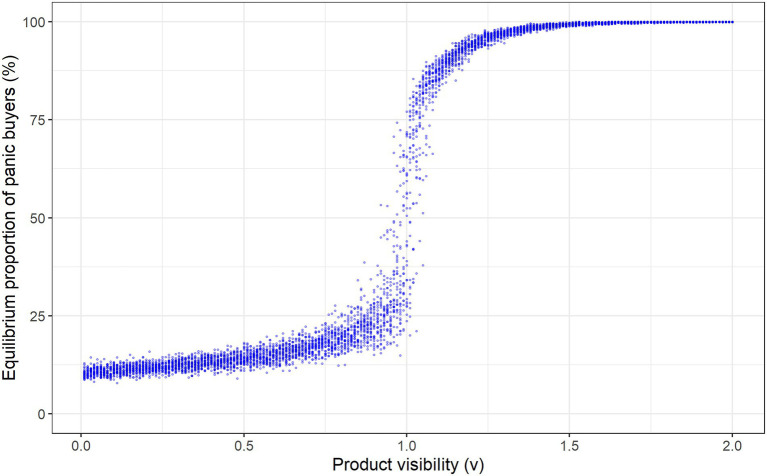
Equilibrium levels of panic buying for products with differing visibility.

The equilibrium level of panic buying depends on the product’s visibility v. To illustrate this relationship, we independently simulate the panic buying process for products with different visibility levels, ranging from low to high ($v\in \left\{0.01,0.02,\ldots ,2\right\}$). Each point in Figure 2 shows the equilibrium proportion of consumers panic buying the product in one simulation of the system; we generate several realizations of the system for each visibility level (each with different randomly drawn thresholds). Figure 2 shows that the size of the cascade depends on the product’s visibility: in equilibrium, only a small proportion of consumers panic buy the low visibility products (e.g., v<0.5), while almost all consumers buy the high visibility products (e.g., v>1.5). This is consistent with some of the behavior observed during the pandemic (e.g., panic buying of high visibility products like toilet paper, but not of lower visibility products like pharmaceuticals).

This panic buying process is analogous to the typical process of innovation diffusion. In the innovation diffusion process, initial adopters of the innovation influence others in their social networks to adopt the innovation, leading to more widespread adoption (in successful cases; Rogers et al., 2019). However, while a wait-and-see approach may be rational for most innovations, in the case of panic buying there is also an element of now-or-never as individuals who do not “adopt” panic buying may miss out on essential products, so we would expect the “innovation” to spread far more quickly.

### Case study 2: Health protective behaviors

Our next case study will focus on protective health behaviors. Health protective behaviors include non-pharmaceutical or behavioral strategies aimed at reducing the risk of negative health outcomes. These are observable behaviors, like panic buying, but require mass actions to be harnessed to be effective, particularly during an event such as the COVID-19 pandemic (Ma and Tsai, 2020). It is important to understand health protective behaviors to improve public health messages and reduce disease transmission (Bavel et al., 2020; West et al., 2020). Yet, early mass compliance or willingness to engage in such behaviors has remained low in the general population during the COVID-19 pandemic (Zeng et al., 2020; Abboah-Offei et al., 2021). For example, despite being relatively low-cost, cheap, and non-invasive, face mask wearing initially remained at low levels in many countries (Quah and Hin-Peng, 2004; Taylor et al., 2009; McIntyre, 2018; Rader et al., 2021).

#### Theoretical explanations for health protective behaviors

Existing Health Psychology models, such as the Health Belief Model (Rosenstock, 1974) and Protection Motivation Theory (PMT; Rogers, 1975) highlight several factors that could facilitate health protective behaviors. These factors include perceived disease threat, perceived disease severity, and perceived efficacy of health protective behaviors, all of which have been shown to facilitate engagement in health protective behaviors during the COVID-19 pandemic (Anaki and Sergay, 2021; Ayre et al., 2021). In the context of face mask use, a recent literature review concluded that increased perceived disease susceptibility was the strongest predictor of these (Sim et al., 2014). Recent studies implementing the PMT framework have found that perceived vulnerability, severity, outcome efficaciousness of health behaviors, and self-efficacy are key predictors of engaging in health-promoting behaviors (Schulz and Hartung, 2020; Ezati Rad et al., 2021; Kowalski and Black, 2021). However, many studies driven by health models focus only on those predictors theorized to be of importance and may exclude other important factors beyond those relating to the individual and their perceptions of their own risk. Other research has shown that social categories can predict whether we perceive a health threat as applicable, which then facilitates our adoption of protective or risky health behaviors (Drury et al., 2021). Newer theoretical models of psychological antecedents of vaccination readiness, such as the 5C model and the 7C model, also highlight the importance of prosocial concerns, including collective responsibility, for vaccination uptake (Betsch et al., 2018; Geiger et al., 2021).

To adopt a relatively new behavior a person must overcome or accept specific individual costs and arrive at a place where these are outweighed by the perceived benefits. Yet, the benefits of many health protective behaviors are not solely individual (Cheng et al., 2020). In the example of mask wearing, individual costs include feeling strange in terms of self-image, difficulties breathing (Pfattheicher et al., 2020), experiencing discomfort and embarrassment (Sim et al., 2014), and overcoming existing habits (Betsch et al., 2020). Yet, wearing a face mask protects from transmitting an infectious disease to others as well as providing some individual safety (Eikenberry et al., 2020). Therefore, people need to weigh social benefits of their actions equally or greater than their own individual outcomes. Recent research has supported this. Face mask use and social distancing have been linked to increased empathy and prosociality (Pfattheicher et al., 2020). Beyond this people also appear to see others as more prosocial when engaging in health protective behaviors, while individuals not wearing face masks are viewed less positively (Betsch et al., 2020).

A study conducted in Germany in 2021 reported that most people engaged in similar health protective behaviors during the peak of the pandemic, but they did so for different reasons: self-protection or group protection (Liekefett and Becker, 2021). Self-protection was typically used as a form of coping with personal anxieties (e.g., personal threat and aversion to uncertainty), while group-protection was facilitated by identifying with a collective goal and perceived societal efficacy for dealing with threat, indicating that group-level efficacy and concern for vulnerable people may be key social facilitators of health protective behaviors.

Much like panic buying, within our social context, social conformity may be a significant driver of health protective behaviors. Recent research has indicated that peer pressure is a key predictor of engaging in health protective behaviors (Nivette et al., 2021) and that mask use is perceived as more acceptable in the presence of other mask wearers (Capraro and Barcelo, 2020; Carbon, 2020). However, in line with SIT the people we are observing may also be critical. Sources considered an ingroup member appear to have greater influence (Jetten, 2020). This may be more important for health behaviors which require more planning and have a different cost–benefit ratio to behaviors such as panic buying. The importance of connection is also highlighted in several other studies which refer to collectivity (Drury et al., 2021), social connectedness or social capital (Putnam, 1995; Chuang et al., 2015). Recent, large-scale global studies also suggest that trust in governments (known to improve connection and capital) predicts engagement in COVID-19-related health protective behaviors (Clark et al., 2020; Wright et al., 2021).

Deeper consideration also needs to be made of an individual and their capacity to adopt health protective behaviors. Dispositional factors such as self-control (i.e., deliberative, effortful, and conscious overriding of impulses) have been related to health protective and risky behaviors (Tangney et al., 2004; Hagger et al., 2009; Keller et al., 2016). Poor emotional regulation and state anxiety have also been shown to predict adoption of health protective behaviors in individuals with high risk perception (Rubaltelli et al., 2020). The importance of habit and its role in the adoption of health behaviors in an ongoing topic of interest (Gardner, 2015; Gardner et al., 2019). Indeed, engaging in healthy behaviors prior to the COVID-19 pandemic predicts greater adherence to health protective behaviors (Nudelman et al., 2021) and past health protective behavior was also related to people’s intention to socially distance in the future (Hagger et al., 2020). Habit research recognizes the importance of people’s inherent behavioral laziness and that much of what we do, we do simply because we did it yesterday and the day before and so on. Habit strength has become recognized as a key driver for many health behaviors (Orbell and Verplanken, 2015). These findings lend support to the idea that past behavior predicts future protection motivation (Hagger et al., 2018). This research suggests that for some individuals, greater effort may be needed which may also represents greater individual cost.

It is interesting that panic buying is so easily triggered when health protective behaviors have taken so much effort to promote at a mass level. Key identified predictors of health protective behaviors include perceived social norms within valued ingroups, identifying with community, a sense of public duty, empathy for at-risk individuals, a sense of ‘being in it together’, social capital, national identification, and sharing values in security and responsibility (Everett et al., 2020; Goldberg et al., 2020; Pfattheicher et al., 2020; Van Bavel and Boggio, 2020; Wolf et al., 2020; Vignoles et al., 2021). Aside from barriers specifically relating to specific behaviors, there are also some general barriers to uptake that need consideration such as psychological reactance (Brehm, 1966; Rosenberg and Siegel, 2018). The tendency to experience reactance has been linked to antisocial and narcissistic personality traits as well as conservative political ideology (Lewing and Caraway, 2019; Irmak et al., 2020). Reactance has been linked to reduced intentions to comply with restrictions (e.g., staying home), but not actual behavior (Krpan and Dolan, 2021). In relation to specific behaviors such as mask wearing, reactance has been linked to anti-mask attitudes, which were in turn linked to actual behavior, i.e., not wearing a face mask (Taylor and Asmundson, 2021). Vague and inconsistent messaging is also likely to have contributed with frequent changes in official government information (Drury et al., 2021). Finally, a reduction of a sense of ‘being in it together’ may reduce health protective behavior engagement at the mass level with evidence that poor linking capital (i.e., lack of trust in governments) is a barrier to practicing behaviors such as mask wearing (Hornik et al., 2021; Keane and Neal, 2021).

#### How does conformity lead to polarization? Modeling behavioral conformity

Conformity can be an important motivator of behavior for health protective behaviors, but if individuals preferentially associate with like-minded people, it could drive polarization rather than consensus or the sense of being in it together. We explore this idea using an agent-based model of social influence, where individuals are situated in a social network and influence (and are influenced by) their social contacts’ behaviors.

**Figure 3:**
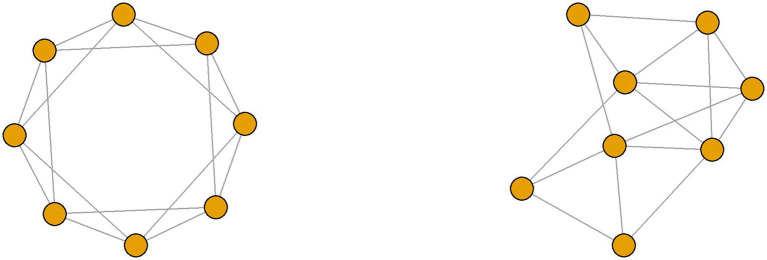
The small world model with *N* = 8, *k* = 4 and rewiring probabilities *p* = 0 (left), giving a ring lattice, and *p* = 0.2 (right).

In the model, we situate individuals in a “small-world network” using the algorithm given by Watts and Strogatz (1998). This involves starting with a ring lattice with N nodes (individuals) and k links (social contacts) per node and then randomly rewiring each link with probability p(see Figure 3 for an example). This allows the generation of networks that exhibit high clustering and low average path length between nodes, a combination observed in many social networks (Jackson, 2010).

We give each agent an initial score representing its propensity to do the behavior which varies between agents (which may be due to different beliefs and/or different personal costs and benefits from the behavior). If this score is positive the agent does the behavior, otherwise, the agent does not. Since in most social networks individuals tend to be linked to similar individuals (Jackson, 2010), we let neighboring nodes (agents) have correlated initial scores (reflecting their similar types). Therefore, when we generate the network, the initial ring lattice gives clusters of similar individuals and the rewiring process (which we control through p) then increases the diversity of individuals’ contacts (as well as creating a small-world structure). We use standard values for p and k in our experiments (Amblard and Deffuant, 2004).

We let the agents be heterogeneous in their levels of conformism, or the degree to which their scores can be influenced by the actions of others. To model this heterogeneity in conformism, we assign each agent a random range within which their score can vary. Agents with wide ranges are relatively conformist (as their scores can be greatly influenced by others’ actions), while agents with narrow ranges are relatively non-conformist.

##### The learning process

The learning process involves individuals updating their scores x_i in response to others’ behavior.

1. Initially, any agent with $x_{i}>0$ does the behavior and all others do not.
2. In each subsequent period, an agent is randomly selected. This agent then:
  - Observes the behavior of one of their (randomly selected) social contacts
  - Updates their score by a small amount θ in the direction of the observed social contact’s behavior, as long as this update keeps the agent’s score within the permissible range
  - Updates their behavior if required (i.e., starts doing the behavior if their score moves from below zero to above zero and vice versa)

**Figure 4:**
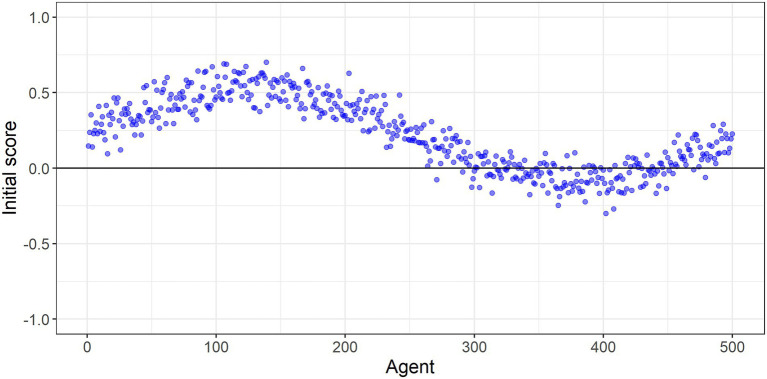
Individuals’ initial scores. Any agent with a score above zero does the behaviour. In the ring lattice the ith agent’s immediate neighbours are the $\left(i-1\right)$th and $\left(i+1\right)$th agents. Since the agents are located on a ring, the neighbours of agent i=1 are agents i=2 and i=500.

In our experiments we generate a small-world network with N=500 agents. Figure 3 shows the agents’ initial scores (or propensities to do the behavior). We set these initial scores such that most agents have moderately positive scores (and are pro-behavior) and a minority of agents have moderately negative scores (and are anti-behavior). Initially, each agent is linked to its 5 immediate neighbors on either side. For example, agent 100 is linked to agents 95–99 and agents 101–105. This generates a social structure in which each agent interacts with agents that have similar scores. We then randomly rewire the links between the agents to increase each agent’s chance of interacting with others that have different views and behavior. For example, the rewiring process could replace one of agent 100’s initial links to an agent with a similar score with a link to an agent with a much different score (e.g., agent 400 in Figure 4). We control this rewiring process with the network’s rewiring probability, which is the probability of replacing one of the initial links between neighboring agents with a link to a random agent. Our experiments test the effect of increasing this rewiring probability (i.e., the degree of mixing between people with diverse views) on the population’s beliefs and behavior.

###### Experiment 1

**Figure 5:**
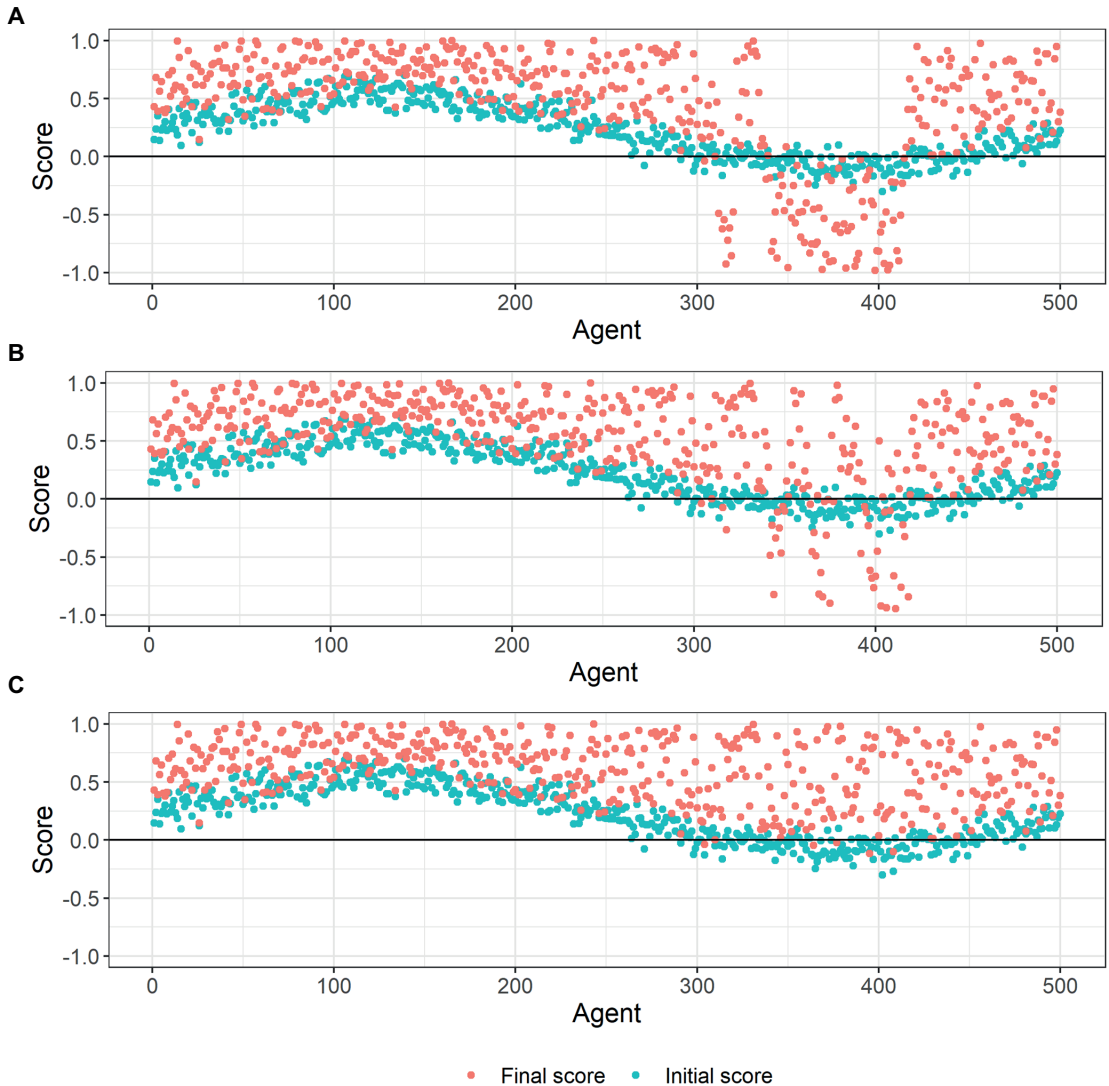
Agents’ initial and final scores in small-world networks with different rewiring probabilities: 0.1 **(A)**, 0.2 **(B)** and 0.3 **(C)**.

We set the rewiring probability at 0.1, such that on average each agent only has one of its initial links randomly rewired. As such, each agent has a very similar view of the behavior (score) to most of its social contacts. We then simulate the social learning process over many time periods. Over time, agents reinforce one another’s behavior, producing polarization, with the vast majority ending up either strongly pro-or anti the behavior, despite starting off from far more moderate positions. This is illustrated in Figure 5A, which shows that the agents’ final scores at the end of the simulation are more polarized than their initial scores. Even though the population is connected to one another within a network, clustering into groups allows the emergence of a strongly anti- group. Such polarised positions are likely to prove resistant to further persuasion or even new information on the benefits or otherwise of the behavior.

###### Experiment 2

We increase the rewiring probability to 0.2, slightly increasing the degree to which agents mix with others that have differing views and behavior. Over time, polarization still emerges, but the strongly anti-group is smaller than in experiment 1, with more agents pulled into the majority pro-group. This is shown in Figure 5B, with fewer agents having negative scores at the end of the simulated social learning process compared to experiment 1.

###### Experiment 3

We increase the rewiring probability to 0.3, again increasing the degree to which agents mix with others that have differing views and behavior. Despite the agents still mostly mixing with those that have similar views, a strong pro-behavior consensus emerges over time. Almost all the agents who were initially anti-behavior end up being pro-behavior due to social influence, with many of them developing strong pro-behavior views. This is illustrated in Figure 5C, which shows that almost all the agents finish with positive scores at the end of the simulation. While the development of a strong pro-behavior consensus is good in terms of adoption of the behavior, it may if anything be too resilient and resistant to change as circumstances change or new information becomes available.

These simulations illustrate how readily conformity can drive behavior among a population with initially mixed, and moderate, views on the desirability of a given behavior. In these simulations, conformity drives behavior through a simple mechanism: each agent incrementally updates their opinion of the behavior to conform with the behavior of their social contacts, which they repeatedly observe over time. Our results show the importance of such non-assortative mixing. Where people tend to associate mostly with others who hold similar views, conformity will drive polarization; in contrast greater mixing of people with diverse views produces consensus, with almost all individuals converging to the same behavior. Conformity can therefore be a strong driver, and triggering conformity is a powerful tool for behavioral change. However, if initial views are mixed and people tend to be most influenced by others with similar views, promoting conformity could result in entrenched polarization. This could lead to some individuals becoming strongly resistant to a given behavior, while those who do adopt it may also become excessively attached to it (making the behavior difficult to change subsequently, if required).

### Case study 3: The collective mind - conspiracy theories

Finally, we consider a mass outcome that is attitudinal rather than an observed behavior. Conspiracy theories (CTs) are typically defined as a shared belief that a group of powerful people are working together towards recognizing a secret plot (van Prooijen and van Vugt, 2018; Goldberg et al., 2020). van Prooijen and van Vugt (2018) suggest that CTs comprise five core components: (1) Assumptions about causal interconnection, (2) Deliberate actions/planning, (3) A coalition working together, (4) A level of threat, and (5) Secrecy.

People who believe in one CT are more likely to believe in other CTs even if they are contradictory (Wood et al., 2012; Lukić et al., 2019). In the US about 1 in 2 people believe in one common medical CT with 1 in 5 believing in 3 or more (Oliver and Wood, 2014). Rates appear consistent but do fluctuate over time (Uscinski and Parent, 2014). Similar rates have also been observed in European countries such as Hungary (Kreko, 2015). There is strong consensus in the literature that CTs can result in multiple negative outcomes for society including promoting further CTs, creating feelings of insecurity, increasing racism, radicalization, interfering with vaccination and regular check-ups, creating mistrust in politicians, promoting science denial, and reducing prosocial behaviors (Vosoughi et al., 2018; Sternisko et al., 2020; Sutton and Douglas, 2020). Recently CTs have been associated with reduced social distancing during the COVID-19 pandemic (Bierwiaczonek et al., 2020). On the positive end, they can call people to action against malevolent powers, change attitudes and increase pressure for greater transparency in processes (Douglas et al., 2019).

#### Theoretical explanations for conspiracy theories

A popular theory to explain the rise of CTs is that they emerge to give people a sense of control over unexpected or complicated events (Kreko, 2015). Themes of control are also evident in literature on panic buying and health protective behaviors. Despite its popularity and face value, a recent meta-analysis of 23 studies found little evidence that CTs resulted from threatened control (Stojanov and Halberstadt, 2020). Other authors suggest that conspiracy beliefs offer a way to look for meaning and purpose at times when communities are facing adversity (van Prooijen and Douglas, 2017). Increases in CTs in times of economic uncertainty support this idea (Kreko, 2015). Therefore, it is possible that this mechanism exists but is not purely underwritten by desires for control.

Kreko (2015) also recognizes an underlying drive for social identity, suggesting shared belief unites people in the ingroup and provides an opportunity to weaken a dominant group. This idea is taken further by van Prooijen and van Vugt (2018) who propose conspiracy theorizing is an evolved psychological mechanism. They suggest as social beings, we have formed several tendencies that serve to bond us with our social group which, in some instances, also creates conflict with those in the outgroup. Indeed, false truths have been spread about people in ethnic minorities throughout history to alienate them (Burdick, 2018) and to raise collective self-esteem. Social identity motives may draw people toward CTs about out-group members, thus protecting their ingroup membership, which is perceived as superior, while uniqueness motives may draw people toward extreme, unusual and non-normative movements (Sternisko et al., 2020).

Despite suggestions that mechanisms underlying CTs may be biologically inbuilt, some individuals appear more prone to this style of thinking than others. A review of personality and conspiracy beliefs suggested good evidence for paranoia, paranoid ideation and schizotypy traits (Goreis and Voracek, 2019). These traits have been long proposed as possible drivers (Burdick, 2018). More recently, research has moved away from psychopathological traits toward more modifiable ones such as preference for extremist ideologies and/or high levels of mistrust (Krouwel et al., 2017). Cognitive styles such as lower levels of analytical thinking and a tendency to tie together unrelated events are also associated with conspiracy theorizing (Goldberg et al., 2020).There are multiple possible triggers for CTs, many which cannot be controlled and none of which are likely to work in isolation. It is most likely that there is specific combination of triggers and the significance and temporal order of these. There has been some suggestion in the post-truth age of politics that powerful entities have purposely tried to create and grow CTs for their own benefit (McIntyre, 2018). Societal crises where existing power structures and norms of conduct need review and revision are likely to be significant drivers (van Prooijen and Douglas, 2017). Significant events, political deception or perpetuation of false information, and power imbalance are other likely triggers.

Mass communication has made some CTs much more salient and has led some to question whether the Internet age, specifically, has been a trigger for conspiracy beliefs. One article tracked the publication of letters published in newspapers in the US between 1890 up to 2010 and scored the amount of conspiracy belief content. They observed two spikes in content, long before the rise of the Internet (Uscinski and Parent, 2014). Furthermore, CTs have existed for centuries particularly in relation to powerful and noted figures (Dianosashvili, 2013).

The Internet may, however, be responsible for changing the nature of some CTs. Inaccurate or misleading information (‘misinformation’) shared on the social media platform, Twitter, has been shown to travel faster and reach a much larger audience than accurate information (Vosoughi et al., 2018). Wood (2013) suggests CTs have moved towards pure denial (claiming fakeness) rather than the development of explanations or description of underlying motives. The internet also gives a louder voice to communities that may have historically been in the fringes. Analysis of social networks online shows how people with climate change denial beliefs cluster and indicates that most people associate with networks with only a single view (Williams et al., 2015). Finally, the Internet is only one part of a changing mass media landscape. Commercial mass news outlets and popular entertainment may also be an independent contributor to conspiracy beliefs (Douglas et al., 2019). As eloquently summarized by Myers and Caniglia (2004) *“Relying on mass media sources for information inevitably produces a simplified, distorted, and incomplete picture of the world around us”* (p. 538).

The internet and mass media underlie a wider cultural shift that may also promote CTs. In 2016, “post-truth” became the Oxford dictionary’s word of the year in a gesture which made concrete a societal and political shift where facts were questioned or ignored and the reputation of science considered biased and motivated (McIntyre, 2018). The post-truth age allows people to weight their existing beliefs equally to fact and is likely a significant driver of conspiracy theories.

#### Attitudes as a contagion: Modeling conspiracy theories

The spread of conspiracy beliefs can be modeled in a similar way to infectious diseases. Here we try applying a modified SIR (Susceptible, Infectious, or Recovered) model to explore how belief in CTs might spread within a population, and the resiliency of such beliefs. In this example, the belief spreads through the population as a result of susceptible individuals coming into contact with those ‘infected’ with the belief. Recovery here is not determined by biology but rather by subsequent interactions with others who do not share the belief (which can occur virtually or face-to-face). However, these interactions are unlikely to occur at random, so we include a parameter that gives non-random mixing of the population, allowing infected people (i.e., believers) to have a greater propensity to interact with other infected people. We let each infected individual’s probability of recovery change over time in response to the reinforcement (or challenging) of their beliefs in interactions with believers and non-believers. In contrast to our model of behavioral conformity, this model considers a narrower process of social influence where only a subset of the population (susceptible and infected individuals) can change their behavior and social influence is asymmetric (infected individuals strongly influence susceptible individuals, while recovered and unsusceptible individuals exert only mild influence on infected individuals).

The model considers the spread of a CT through a population of N=1,000individuals across time periods t=0,1,…,T. Most people are unlikely to ever believe any given CT. Here we assume that 10% of the population ($S_{0}=100$ individuals) are susceptible to believing the theory, with 10% of those ($I_{0}=10$) believing it at the start of the simulation. In each period, two individuals are matched *via* the following process:

- Individual i is randomly drawn from the population
- If individual i is infected, they are matched with another infected individual with probability m=0.5; otherwise, they are matched to a random individual. The probability m represents the fact that believers are more likely to discuss the CT with other believers (discussed further below)
- If individual i is uninfected, they are matched with another individual at random

The two matched individuals (i and j) then have an interaction where they discuss their views on the CT. This leads to the following state updates:

- If one of the individuals is susceptible and the other is infected, then the susceptible individual becomes infected
- If one of the individuals is not susceptible (including recovered) and the other is infected, then the infected individual recovers with probability $p_{\mathrm{it}}$, i.e., the believer in the CT has their views challenged and changes their mind. We set the initial probability of recovery at $p_{i0}=0.05$ to make recovery somewhat uncommon
- If both individuals are infected, then their probabilities of recovery are reduced to $p_{\mathrm{it}}=0.9p_{i,t-1}$ and $p_{jt}=0.9p_{j,t-1}$ respectively, i.e., the two believers reinforce each other’s beliefs, reducing their chances of subsequently abandoning the beliefs.

**Figure 6:**
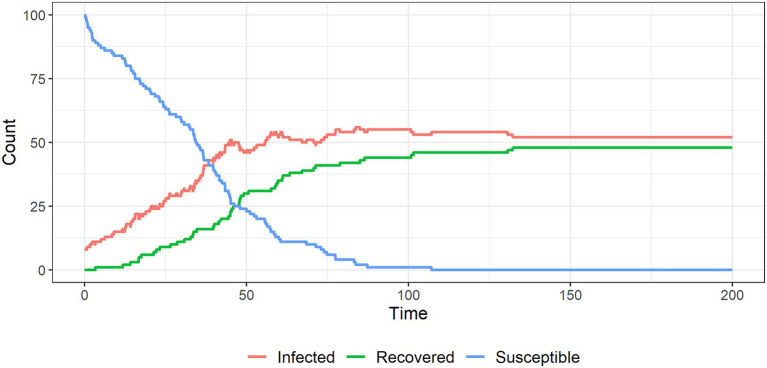
The spread of a conspiracy theory in a population; around half of the individuals remain ‘infected’ with the conspiracy belief in this baseline scenario.

**Figure 7:**
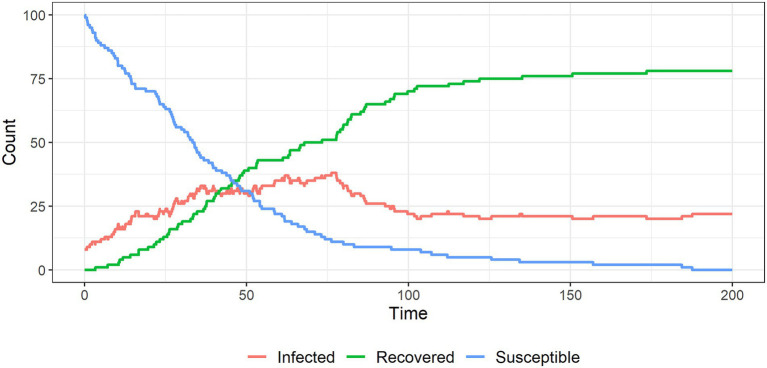
The spread of a conspiracy theory in a population, in which it is less likely that believers will be able to interact with other believers, resulting in a lower equilibrium number of ‘infected’ individuals.

**Figure 8:**
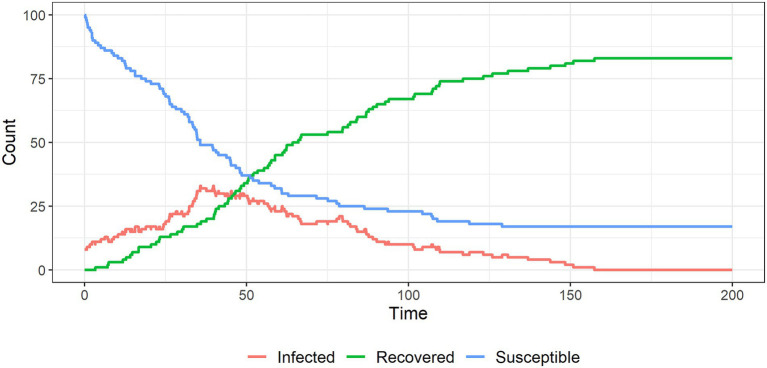
The spread of a conspiracy theory infection through a population, where believers cannot reinforce each other’s beliefs. The conspiracy theory dies out under these settings.

**Figure 9:**
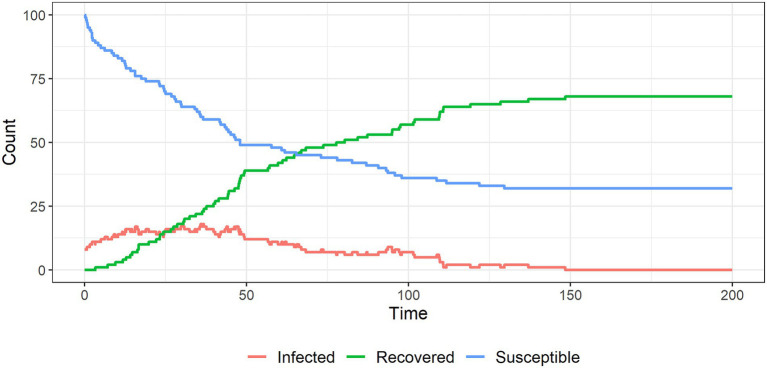
The spread of a conspiracy theory infection through a population, with cumulative social influence from non-susceptible individuals on susceptible individuals. Again, the CT dies out as believers are convinced of their error.

Figures 6–9 show the results of simulations exploring the effects of agent behavior on aggregate belief in the CT. These figures show the counts of susceptible, infected, and recovered individuals over time (where time is defined as the mean number of interactions per individual). Simulation 1 (Figure 6) uses the baseline parameter values above. Simulations 2–4 (Figures 7–9) each alter one parameter value from the baseline settings.

In simulation 1 (Figure 6) all susceptible individuals become infected as the population interacts. The system reaches an equilibrium in which about half of the infected individuals remain infected forever: these individuals have reinforced each other’s beliefs to the point where their probabilities of recovery are infinitesimally small. The remaining infected individuals recover due to interactions with unsusceptible individuals.

In simulation 2 (Figure 7) we reduce the probability of an infected individual interacting with another infected individual (reducing m from 0.5 to 0.2). This reduces the degree to which infected individuals reinforce each other’s views and increases their exposure to unsusceptible non-believers who challenge their views. Figure 7 shows that the system reaches an equilibrium in which fewer individuals remain infected compared to the baseline.

In the baseline setting, when two infected individuals interact, they reinforce each other’s beliefs and become less likely to abandon them. This is represented by reducing their probabilities of recovery. In simulation 3, we let the probabilities of recovery be constant to remove this reinforcement mechanism. This causes the CT to burn out over time (Figure 8). Therefore, some level of belief reinforcement is required to produce a long-run equilibrium in which individuals believe in the CT (otherwise interactions with unsusceptible individuals eventually cause infected individuals to recover).

In simulation 4 (Figure 9), we let the unsusceptible individuals exert cumulative social influence on the infected individuals. To do this, we increase the infected individual’s probability of recovery by 10% following an interaction with an unsusceptible individual (e.g., from 5% initially to 5.5% after one interaction with an unsusceptible individual and so on). Figure 9 shows that this cumulative social influence on infected individuals causes belief in the CT to burn out quickly and limits overall infection.

These simulations highlight the importance of mixing between people who are susceptible to, believe on or are resistant to an extreme belief such as a conspiracy belief. Digital platforms may increase the rate at which susceptible individuals encounter CTs due to increasing the spread of information. They may also make it easier for believers to limit their interactions to those who share their beliefs, reducing the likelihood of recovery (i.e., echo chambering).

## Discussion

Throughout the case studies examined, the utility of Social identity theory (SIT; Tajfel, 1978) has emerged. The formation of ingroups and outgroups appears to lead to several psychological phenomena, the most prominent of which are ingroup favoritism (acting in ways to assist the group that is a person identifies with), and outgroup bias (disliking, generalizing and not being helpful to those outside of the core group). These groups are fluid and self-defined. For example, the ingroup may be supporters of a particular sporting team while supporters of others teams are the outgroup; however, if simply being a sports fan is made salient, the ingroup may be supporters of football teams while the outgroup is people who do not identify with a team (Levine et al., 2005). Given that multiple self-schemas and concepts can exist within a person, identifying which is most salient in each situation is important to predict future mass behaviors. It has been suggested that schemas are core or central to our self-concept and these are the most elaborated and valued (Markus and Wurf, 1987) and this seems like a good place to start assessing the strength of ingroup identification across self-concepts.

Our ability to work across disciplines has provided novel insights. This may at times come at the sacrifice of depth, but has facilitated an understanding of key, repeated themes. The mathematical models provided a way to explore the relationship between individual behavior and collective outcomes for three different mass behaviors. The agents in these models followed simple decision-making heuristics and the models showed how these heuristics could generate some of the collective outcomes observed during the COVID-19 pandemic. We chose to use three different models to focus on the key elements of individuals’ behavior in the context of each mass behavior. The models showed that different behavioral processes could lead to similar aggregate outcomes, such as consensus and polarization. Building on these models by including more psychological variables and incorporating more detailed simulations could result in a useful method to predict future mass behaviors (Musse et al., 2021).

Several other themes are repeated throughout the case studies:

### Stress and the need for clarity or control

Stress and the need to restore control is the most popular explanation underlying observable and attitudinal mass behaviors, albeit the mechanism appears more complicated across the case studies examined. Negative emotions appear to trigger a heightened focus on external cues (Fiedler, 2001), which then makes us more vulnerable to social cues and other biologically determined social drivers. This extends to our thinking, it appears that negative feelings drive us to a place of comfort (using existing beliefs to explain events) or to a search for meaning. For example, fear and perceived risk can be strong drivers of health behaviors, but too much fear without a balance of feelings of control can result in counter-intuitive actions or an increase in conspiracy beliefs. It is also important to remember that while some significant social events can create mass changes in affect (e.g., decreases in wellbeing during COVID-19), fear is also constructed at an individual level based on an individual’s actual risk, awareness, and level of concern (e.g., in the case of mask wearing).

### Individual propensity

If we take the view that behaviors are biologically predisposed, the obvious question is, do we all do the same behaviors to the same extent? The answer is no. Certain individuals may be more predisposed to engage in mass behaviors than others. There is no universality to these characteristics; they vary according to the example considered. Someone with high levels of worry may be more likely to seek comfort by enacting health behaviors or adopting conspiracy beliefs. That said, there are two elements that are both state and trait that appear to underlie mass behaviors: trust and social connection. People who are naturally distrusting are more prone to psychological reactance, conspiracy beliefs, and less likely to follow advice about health protective behaviors. The same is true of those who feel a low sense of connection to their community. Another individual characteristic that may be important is education level; most likely mitigated through increasing analytical thinking skills, which seems to protect against negative mass behaviors and promote positive behaviors.

### Trust

Trust in governments and other officials appears to be a common facilitator, and distrust a barrier, to engaging in multiple crowd behaviors. Distrust appears to be an issue that has become particularly salient in times of post-truth, mass media and social media use. Trust also occurs at macro and micro-levels and is something that is hard to establish and easy to break. Trust in larger institutions and government needs broader systemic changes whereas trust in individuals can be built in more simple ways.

### Connection with others

The aim to increase our social connections with people that we identify with on some level appears to be an important driver of mass behaviors, including health protective behaviors, as well as less positive behaviors such as conspiracy theories. Those with less social connections are more likely to adopt conspiracy beliefs. However, social connections with certain groups of people are likely to perpetuate and cement these beliefs through creating a well-defined us (ingroup) versus them (outgroup). Connection also includes perceptions of belonging and value such as collective self-esteem.

### External cues

Some of the mechanisms considered suggest that certain events trigger a heightened awareness or sensitivity to external cues and information. These appear to be present across multiple examples considered. SIDE theory proposes that people in groups become more sensitive to external information about the group and less driven by their own individual identity (Reicher et al., 1995).

Thus, it may be important in the future to consider how to reduce a focus on external cues, particularly if simple cues promote undesirable behaviors such as panic buying. Mood may be an important variable in this relationship; while not particularly easy to change on a mass level, understanding the consequences of certain moods is critical to mitigation. Another external cue is visibility. People more focused on their external environment tend to be highly driven by visual cues. We see this in the example of purchasing bulky toilet paper packets. This tendency could equally be used to promote behaviors like mask wearing where they are made more publicly visible.

### Societal influences

There is good evidence that there are certain, significant events which in themselves are likely to be drivers of mass behavior. This includes political structures but also external events such as pandemics and natural disaster. Some of these can be controlled, while others cannot. The structures put in place to manage them and the way they are implemented can be controlled. The way we connect socially is also subject to recent change with the introduction of social media and the Internet which adds a new variable to traditional social psychological models that include interactions between people primarily in person. Health departments have relied on these media to share critical information which has allowed the sharing of messages to broader groups of people but also encouraged what the World Health Organization refer to as an “infodemic” (Zarocostas, 2020). Media adds a complex vector to a number of interactions and needs much deeper integration.

### The good with the bad

Not all mass behavior is bad. In the context of COVID-19, mutual aid groups have been established but these may decline as people run out of emotional or physical resources, the government steps in to assist, or people perceive that enough money has been donated and their donation will no longer matter (Drury et al., 2021). Other research on COVID-19 suggests it is a complex situation involving micro- and macro-level factors, such as economic uncertainty and health disaster aspects, both of which were shown to negatively impact upon helping behaviors (Shoss et al., 2021). Prosocial mass behavior in the form of donations following disaster has been shown to depend largely on increased media exposure (Zagefka and James, 2015), which aligns with the social identity approach such that people are more likely to donate to others perceived to be members of their ingroup. Promoting saliency of a common identity may promote prosocial behavior toward outgroup members. However, several factors may hinder or extinguish prosocial behaviors. These include the reinstatement of negative inter-group relations, lack of a common fate or a unifying factor (e.g., widespread exposure; Ntontis et al., 2020). It is important to understand mass behaviors to better harness them for benefit. Based on the case studies explored, it is crucial to build trust at micro- and macro-levels, increase analytical thinking and create or leverage social capital.

### Putting it into practice

Understanding the unique and common drivers of mass behavioral responses to such events helps us better detect crowd behaviors, and in turn, develop strategies to mitigate responses to similar future events. These mitigation strategies may involve those aimed at increasing positive responses (e.g., prosocial donations to disaster victims or wearing face masks) or minimizing negative responses (e.g., panic buying or disregarding health advice). Once crowd behaviors are unleashed, they are difficult to reign in suggesting that mitigation strategies should be proactive rather than reactive, which specific mitigation strategies are likely to be effective and able to be deployed on large-scale in the long-term requires further evaluation.

### Conclusion

We have illustrated the complexities of mass behaviors, namely, that they can be both negative and positive in nature yet underpinned by similar underlying constructs. A remaining question is, what combination of factors leads to more positive or negative crowd behaviors driven by a large-scale social trigger whether an isolated (i.e., a natural disaster) or ongoing event (i.e., infectious disease pandemic)? Another question is, whether the quality or quantity of our social identities translates to positive or negative outcomes? This ties in to the role of mass and social media. Future research aimed at understanding, detecting, and mitigating mass behaviors will likely benefit from integrating descriptive theoretical frameworks, statistical modeling techniques, and evidence from empirical investigations to develop measures and behavior change techniques for harnessing the ‘madness’ of crowd behaviors for societal benefit.

## Author contributions

EB led the study conceptualization and writing—original manuscript preparation, reviewing, and editing. NK contributed to the study design and manuscript preparation. AR contributed to the design, manuscript preparation, formal analysis, and visualization. DE contributed to the formal analysis and visualization. All authors contributed to the article and approved the submitted version.

## Conflict of interest

The authors declare that the research was conducted in the absence of any commercial or financial relationships that could be construed as a potential conflict of interest.

## Publisher’s note

All claims expressed in this article are solely those of the authors and do not necessarily represent those of their affiliated organizations, or those of the publisher, the editors and the reviewers. Any product that may be evaluated in this article, or claim that may be made by its manufacturer, is not guaranteed or endorsed by the publisher.
